# Microwave-Assisted Rapid Synthesis of Reduced Graphene Oxide-Based Gum Tragacanth Hydrogel Nanocomposite for Heavy Metal Ions Adsorption

**DOI:** 10.3390/nano10081616

**Published:** 2020-08-18

**Authors:** Bhawna Sharma, Sourbh Thakur, Djalal Trache, Hamed Yazdani Nezhad, Vijay Kumar Thakur

**Affiliations:** 1 School of Chemistry, Faculty of Sciences, Shoolini University, Solan, Himachal Pradesh 173229, India; sharmabhanusln@gmail.com; 2 Center for Computational Materials Science, Institute of Physics, Slovak Academy of Sciences, 84511 Bratislava, Slovakia; 3 UER Chimie Appliquée, Ecole Militaire Polytechnique, Bordj El-Bahri, Algiers 16046, Algeria; djalaltrache@gmail.com; 4 Department of Mechanical Engineering and Aeronautics, City University of London, London EC1V0HB, UK; hamed.yazdani@city.ac.uk; 5 Biorefining and Advanced Materials Research Center, Scotland’s Rural College (SRUC), Kings Buildings, West Mains Road, Edinburgh EH9 3JG, UK; 6 Department of Mechanical Engineering, School of Engineering, Shiv Nadar University, Uttar Pradesh 201314, India

**Keywords:** reduced graphene oxide, gum tragacanth, hydrogel, hydrogel composite, mercury ion, chromium ion, reusability

## Abstract

Reduced graphene oxide (RGO) was synthesized in this research via Tour’s method for the use of filler in the hydrogel matrix. The copolymerization of *N*,*N*-dimethylacrylamide (DMA) onto the gum tragacanth (GT) was carried out to develop gum tragacanth-cl-*N*,*N*-dimethylacrylamide (GT-cl-poly(DMA)) hydrogel using *N*,*N*’-methylenebisacrylamide (NMBA) and potassium persulfate (KPS) as cross-linker and initiator correspondingly. The various GT-cl-poly(DMA) hydrogel synthesis parameters were optimized to achieve maximum swelling of GT-cl-poly(DMA) hydrogel. The optimized GT-cl-poly(DMA) hydrogel was then filled with RGO to form reduced graphene oxide incorporated gum tragacanth-cl-*N*,*N*-dimethylacrylamide (GT-cl-poly(DMA)/RGO) hydrogel composite. The synthesized samples were used for competent adsorption of Hg^2+^ and Cr^6+^ ions. Fourier transform infrared, X-ray powder diffraction, field emission scanning electron microscopy, energy-dispersive X-ray spectroscopy were used to characterize the gum tragacanth-cl-*N*,*N*-dimethylacrylamide hydrogel and reduced graphene oxide incorporated gum tragacanth-cl-*N*,*N*-dimethylacrylamide hydrogel composite. The experiments of adsorption-desorption cycles for Hg^2+^ and Cr^6+^ ions were carried out to perform the reusability of gum tragacanth-cl-*N,N*-dimethylacrylamide hydrogel and reduced graphene oxide incorporated gum tragacanth-cl-*N,N*-dimethylacrylamide hydrogel composite. From these two samples, reduced graphene oxide incorporated gum tragacanth-cl-*N,N*-dimethylacrylamide exhibited high adsorption ability. The Hg^2+^ and Cr^6+^ ions adsorption by gum tragacanth-cl-*N,N*-dimethylacrylamide and reduced graphene oxide incorporated gum tragacanth-cl-*N,N*-dimethylacrylamide were best suited for pseudo-second-order kinetics and Langmuir isotherm. The reported maximum Hg^2+^ and Cr^6+^ ions adsorption capacities were 666.6 mg g^-1^ and 473.9 mg g^-1^ respectively.

## 1. Introduction

Continuous industrialization leads to the excessive release of toxic pollutants into various sources of water. The heavy metal poisoning of water has now become a pandemic concern due to its dangerous impacts to human health because these pollutants are non-degradable, poisonous, cancer-causing agent and are hard to separate from water [1]. For metal uptake from water, several approaches have been established, among them, treatment via adsorption is the most appealing one. Adsorption technology is widely used for removing pollutants due to its simple operation, cost and easy implementation [2]. Specifically, hydrogels based on biopolymer have now become very useful in adsorptive wastewater treatment [3]. Gum tragacanth based hydrogel is highly adsorptive because of the presence of hydroxyl (–OH) and carboxyl (–COOH) groups [4,5]. It is a renewable, cost-effective and environmentally friendly polysaccharide that can be easily polymerized to form cross-linked structures [6,7,8].

Gum tragacanth is commonly found in the sap of different legumes in the Middle East. The biological source of gum tragacanth is a plant named *Astragalus gummifer*. It is a complex mixture of polysaccharides including bassorin and tragacanthin units. When mixed with water, gum tragacanth produces a colloidal hydrosol. The bassorin unit can (composed of 60–70% of the compound) swells to form a gel [9]. Mallakpour et al. reported the glutaraldehyde cross-linked gum tragacanth/CaCO_3_ hydrogel composite as an adsorbent for the abstraction of Pb^2+^ ion [10]. Moghaddam et al. synthesized methoxyl gum tragacanth-glutamic acid/polyacrylamide hydrogel via electron beam radiations as an adsorbent for trapping uranium ions from toxic uranium solution [11].

The adsorption and stability of hydrogel can be improved by using reduced graphene oxide as filler in the hydrogel matrix. Reduced graphene oxide (RGO) can result in high C/O with better mechanical strength [12]. The reduced graphene oxide is partially decorated with an oxygen-rich functional group that acts as active sites for interaction. The high RGO surface, large porosity and defect sites are the features that help pollutants adsorption [13]. Sahraei et al. reported adsorption of Cr^6+^ metal using chitosan/reduced-graphene oxide/montmorillonite composite hydrogel. The composite hydrogel showed maximum Cr^6+^ absorption of 87.03 mg g^−1^ [14]. Zhuang et al. synthesized molybdenum disulfide/RGO hydrogel as an adsorbent for mercury ions removal [15].

The synthesized gum tragacanth-cl-*N,N*-dimethylacrylamide (GT-cl-poly(DMA)) and reduced graphene oxide incorporated gum tragacanth-cl-*N,N*-dimethylacrylamide (GT-cl-poly(DMA)/RGO) hydrogel composite were efficient in adsorption of Hg^2+^ and Cr^6+^ as compared to previously reported adsorbents in the literature (Table 1). This was due to the perfect combination of reduced graphene oxide (RGO), gum tragacanth (GT), and *N,N*-dimethylacrylamide (DMA) led to the presence of many –OH, –NH_2_ and –COOH hydrophilic groups. The gum tragacanth-cl-*N,N*-dimethylacrylamide hydrogel and reduced graphene oxide incorporated gum tragacanth-cl-*N,N*-dimethylacrylamide hydrogel composite exhibited the highest removal capacity of 625 mg g^−1^ and 666.6 mg g^−1^ respectively for Hg^2+^. Similarly, for Cr^6+^, removal capacities were 401.6 mg g^−1^ and 473.9 mg g^−1^ respectively.

The previously reported works (Table 1) have not comprehensively considered the factors responsible for the high adsorption capability of the adsorbent. In this work, we achieved a better adsorption capacity of 666.6 mg g^−1^ and 473.9 mg g^−1^ for mercury and chromium ions within less time using a low adsorbent dose. Specifically, prepared reduced graphene oxide incorporated gum tragacanth cross-linked poly *N,N*-dimethylacrylamide hydrogel composite shows a very high adsorption percentage of 99% for mercury metal ion under optimal conditions (adsorbent dose = 0.035 g and time = 270 min, T = 25 °C, the concentration of mercury solution = 20 ppm) which means it is highly efficient for mercury adsorption. Also, compared to recently reported studies, we are able to synthesize our adsorbents in very short period (90 s) with high swelling percentage (Table 2) using microwave radiations. This is one of the key points where our synthesis part shows novelty. Hence, we developed the simple and fast synthetic route for the preparation of efficient, sustainable and eco-friendly graphene oxide incorporated gum tragacanth-cl-*N,N*-dimethylacrylamide hydrogel with high adsorption rate for heavy metal ions.

In this work, we developed first-time gum tragacanth-cl-*N,N*-dimethylacrylamide hydrogel and reduced graphene oxide incorporated gum tragacanth-cl-*N,N*-dimethylacrylamide hydrogel composite for adsorption of Hg^2+^ and Cr^6+^. The RGO was synthesized from graphite and incorporated in GT-cl-poly(DMA) hydrogel matrix to increase the adsorption efficiency. The sorption study was explained by kinetic and isotherm models. The effect of pH, adsorbent dose and RGO loading on adsorption were performed. The gum tragacanth-cl-*N,N*-dimethylacrylamide hydrogel was systematically designed based on swelling. The adsorbed samples were desorbed successfully by using 0.1 M HNO_3_ and used further for adsorption experiments. 

## 2. Materials and Methods

### 2.1. Materials

Gum tragacanth (GT), (molecular weight = 8.4 × 10^5^ g mol^−1^), potassium persulfate (KPS), *N,N*’-methylenebisacrylamide (NMBA), *N,N*-dimethylacrylamide (DMA) were purchased from Sigma-Aldrich (Sigma Aldrich Chemicals Pvt. Ltd., Delhi, India). Graphite powder, H_3_PO_4_, KMnO_4_, H_2_O_2_ and concentrated H_2_SO_4_ (98 wt %) were obtained from LOBA-Chemie (Loba Chemie Pvt. Ltd., Tarapur, Maharashtra, India). Hg^2+^ and Cr^6+^ ions solutions were obtained by dissolving mercury chloride (Sigma Aldrich Chemicals Pvt. Ltd., Delhi, India) and potassium chromate (Sigma Aldrich Chemicals Pvt. Ltd., Delhi, India) reagents in distilled water.

### 2.2. Synthesis of Reduced Graphene Oxide (RGO)

For the synthesis, a mixture of 50 mL concentrated H_2_SO_4_ and 5.5 mL of H_3_PO_4_ was taken in a beaker. Then, 1.0 g of graphite powder and KMnO_4_ (4.0 g) were added to the mixture after maintaining 10 °C under magnetic stirring in ice bath. The mixture was heated to 35 °C governed by a water bath. After 2 h of stirring, the reaction mixture was sonicated 10 times with the help of ultrasonicator. To stop the reaction, deionized water (200 mL) and 1 M NaOH solution were added dropwise to maintain the pH = 6 of solution mixture. The reaction process was then followed with the addition of H_2_O_2_ (15 mL) which led to a change in suspension color to yellow. The mixture was kept overnight. Thereafter, the solution of ascorbic acid (0.227 mol L^−1^) was added dropwise under magnetic stirring at 95 °C. In this step, the color of the solution changes slowly from greenish-yellow to black. Finally, the mixture was allowed to settle down for 1 h. Black precipitates were formed which were then centrifuged and washed several times using ethanol.

### 2.3. Synthesis of Gum Tragacanth-cl-N,N-dimethylacrylamide (GT-cl-poly(DMA)) Hydrogel

Microwave-assisted copolymerization method was used in the synthesis of gum tragacanth-cl-*N,N*-dimethylacrylamide hydrogel [5]. In a typical reaction, 0.5 g of gum tragacanth (GT) (in 11 mL of deionized water) was taken in 50 mL beaker, stirred until GT was uniformly mixed with distilled water. After this, KPS (10 × 10^−1^ mol L^−1^) and NMBA (5.8 × 10^−1^ mol L^−1^) were added into the GT solution. Magnetic stirring was continued to get a homogenous mixture and then 4.4 × 10^−1^ mol L^−1^ of DMA was added in this mixture. The solution mixture was placed under microwave radiations for 90 s to generate active radicals needed for the initiation of the polymerization reaction. The prepared gel was washed using acetone and dried inside the preheated (50 °C) hot air oven for 24 h.

### 2.4. Synthesis of Reduced Graphene Oxide Incorporated Gum Tragacanth-cl-N,N-dimethylacrylamide (GT-cl-poly(DMA)/RGO) Hydrogel Composite

**Scheme 1:**
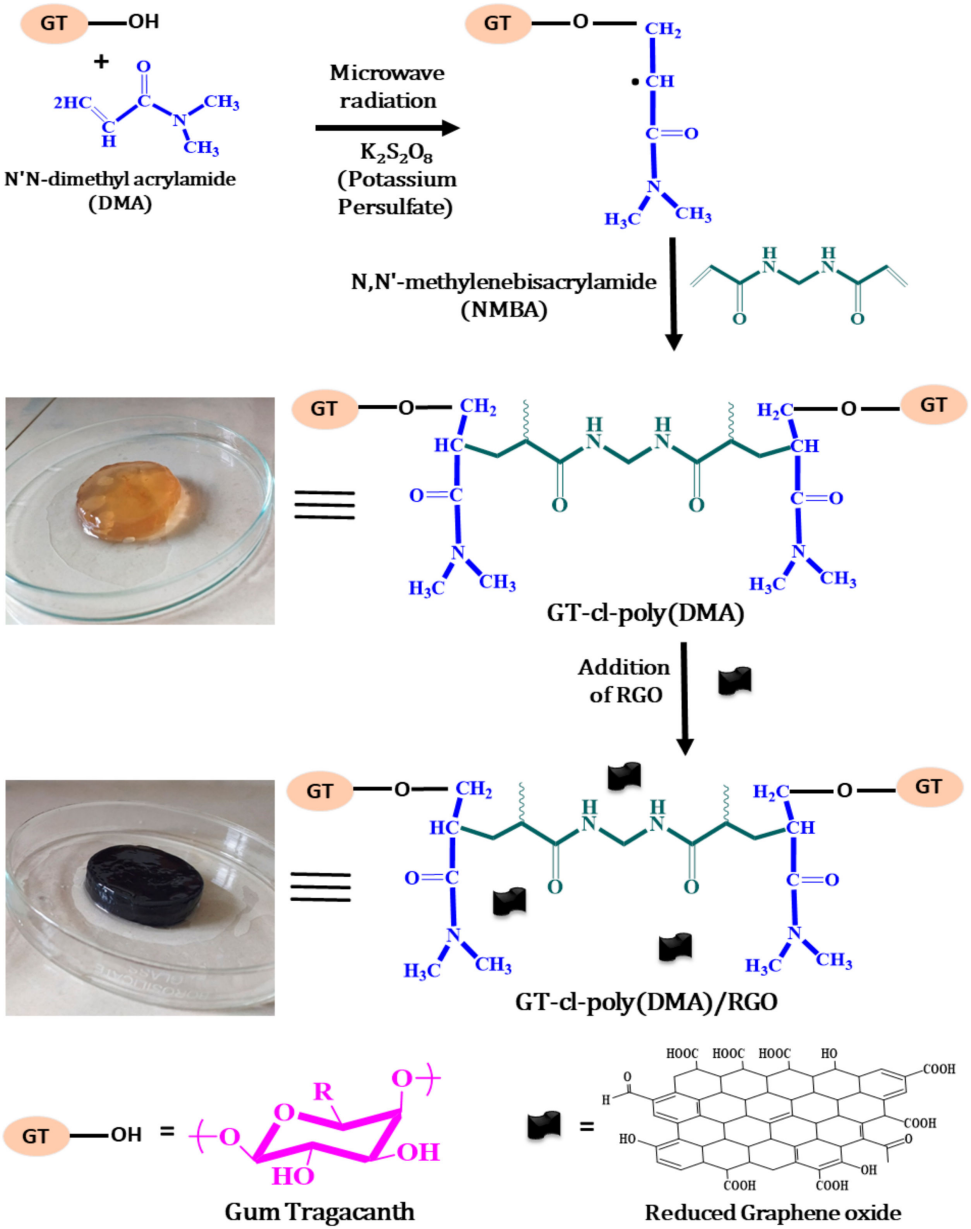
General scheme for the synthesis of gum tragacanth-cl-*N,N*-dimethylacrylamide hydrogel and reduced graphene oxide incorporated gum tragacanth-cl-*N,N*-dimethylacrylamide hydrogel composite.

For the preparation of reduced graphene oxide incorporated gum tragacanth-cl-*N*,*N*-dimethylacrylamide hydrogel composite, 0.5 g of GT was added in a solution of RGO (0.005–0.025 g in 11 mL of deionized water). Physical agitation was applied until the mixture becomes homogenously uniform. Thereafter, the mixture was treated similarly following the procedure in Section 2.3. The incorporation of RGO was confirmed physically by monitoring the color change from light orange to black, as presented in Scheme 1. The optimized quantities that are used in the preparation of hydrogels are given in Table 3.

### 2.5. Characterization

Fourier transform infrared spectra of GT, RGO, gum tragacanth-cl-*N,N*-dimethylacrylamide hydrogel and reduced graphene oxide incorporated gum tragacanth-cl-*N,N*-dimethylacrylamide hydrogel composite were measured through L1600312 spectrum TWOLITA/ZnSe FTIR spectrophotometer (Agilent Technologies, Santa Clara, CA, USA). Field emission scanning electron microscopy images were collected at different resolutions from a Nova Nano SEM-450 FESEM (JFEI, USA (S.E.A.), Hillsboro, ORE, USA). X-ray powder diffraction was collected from a SmartLab 9 kW rotating anode x-ray diffractometer (Rigaku Corporation, Tokyo, Japan).

### 2.6. Swelling Study

Various parameters such as initiator concentration (KPS) solvent volume, time, cross-linker concentration (NMBA), microwave power, monomer concentration (DMA) and amount of RGO were optimized to obtain the maximum swelling percentage. The swelling percentages of gum tragacanth-cl-*N,N*-dimethylacrylamide and reduced graphene oxide incorporated gum tragacanth-cl-*N,N*-dimethylacrylamide hydrogels in deionized water were examined for a fixed period of 16 h. The pre-weighed dry piece of the sample was added in 50 mL of distilled water for 16 h. Then, swelled hydrogel was weighed. The swelling percentage was calculated using Equation (1) [28]:

(1) $$Swelling\;\%=\frac{W_{s}\;-{\;W}_{d}}{W_{d}}\times 100$$

where, W_s_ = weight of swelled gum tragacanth-cl-*N,N*-dimethylacrylamide hydrogel, W_d_ = weight of dry gum tragacanth-cl-*N,N*-dimethylacrylamide hydrogel.

### 2.7. Adsorption of Hg^2+^ and Cr^6+^

The batch adsorption analyses were performed in 150 mL beaker using adsorption shaker (200 rpm) at pH of 5.5 and 3.5 for Hg^2+^ and Cr^6+^ removal respectively. For more illustration, 0.010–0.070 g of adsorbents were used in Hg^2+^ and Cr^6+^ ions solution (50 mL, 20 ppm) at 25 °C for fixed period. After adsorption, the mixture was filtered to determine the concentration of Hg^2+^ and Cr^6+^ ions using 1,3-diphenylcarbazide method [14]. The concentration of adsorbed Hg^2+^ and Cr^6+^ was calculated by evaluating the absorbance of heavy metal ion solution using UV-Vis spectrophotometer at 532 nm and 370 nm respectively. The amount of adsorbed Hg^2+^ and Cr^6+^ was calculated by Equation (2) [28]:

(2) $$q_{e}=\frac{(C_{o}-C_{e})V}{M}$$

where q_e_ = equilibrium gum tragacanth-cl-*N,N*-dimethylacrylamide and reduced graphene oxide incorporated gum tragacanth-cl-*N,N*-dimethylacrylamide adsorption capacity, C_o_ = initial Hg^2+^ and Cr^6+^ concentration (mg L^−1^), C_e_ = equilibrium Hg^2+^ and Cr^6+^ concentration (mg L^−1^), V = volume (L) of Hg^2+^ and Cr^6+^ ion solution, M = weight (g) of gum tragacanth-cl-*N,N*-dimethylacrylamide and reduced graphene oxide incorporated gum tragacanth-cl-*N,N*-dimethylacrylamide hydrogels. Adsorption-desorption analyses were conducted by using 0.1 M HNO_3_. We have optimized the HNO_3_ concentration (0.02 M, 0.04 M, 0.06 M, 0.08 M, 0.1 M, 0.12 M) for desorption experiments. The maximum adsorption-desorption rate was found at optimized 0.1 M HNO_3_. The Hg^2+^ and Cr^6+^ loaded GT-cl-poly(DMA) hydrogel and GT-cl-poly(DMA)/RGO hydrogel composite were desorbed by using 100 mL of 0.1 M HNO_3_ followed by neutralization with 0.1 M NaOH. Finally, the desorbed adsorbent was washed by distilled water and dried at room temperature for further adsorption of Hg^2+^ and Cr^6+^. 

## 3. Results

### 3.1. Mechanism for Synthesis of Gum Tragacanth-cl-N,N-dimethylacrylamide Hydrogel

In this work, gum tragacanth-cl-*N,N*-dimethylacrylamide hydrogel and reduced graphene oxide incorporated gum tragacanth-cl-*N,N*-dimethylacrylamide hydrogel composite were prepared by radical copolymerization of DMA and GT in the presence radical initiator (KPS) and the cross-linking action of NMBA. Under microwave radiations, KPS was decomposed and radical ions were generated [25]. These primary free radicals led to the generation of DMA monomer radical (through addition reaction with KPS) and GT alkoxy radical (through abstraction of hydrogen by KPS). The grafting of DMA radical and GT alkoxy radical was carried through radical copolymerization reaction (Scheme 1). The crosslinker NMBA led to the cross-linkages between different chains to facilitate the construction of three-dimensional gum tragacanth-cl-*N,N*-dimethylacrylamide hydrogel polymeric network. Finally, the dispersion of RGO led to the generation of reduced graphene oxide incorporated gum tragacanth-cl-*N,N*-dimethylacrylamide hydrogel composite.

### 3.2. Optimization of Swelling for Reduced Graphene Oxide Incorporated Gum Tragacanth-cl-N,N-dimethylacrylamide Hydrogel Composite

#### 3.2.1. Initiator (KPS) Concentration

**Figure 1:**
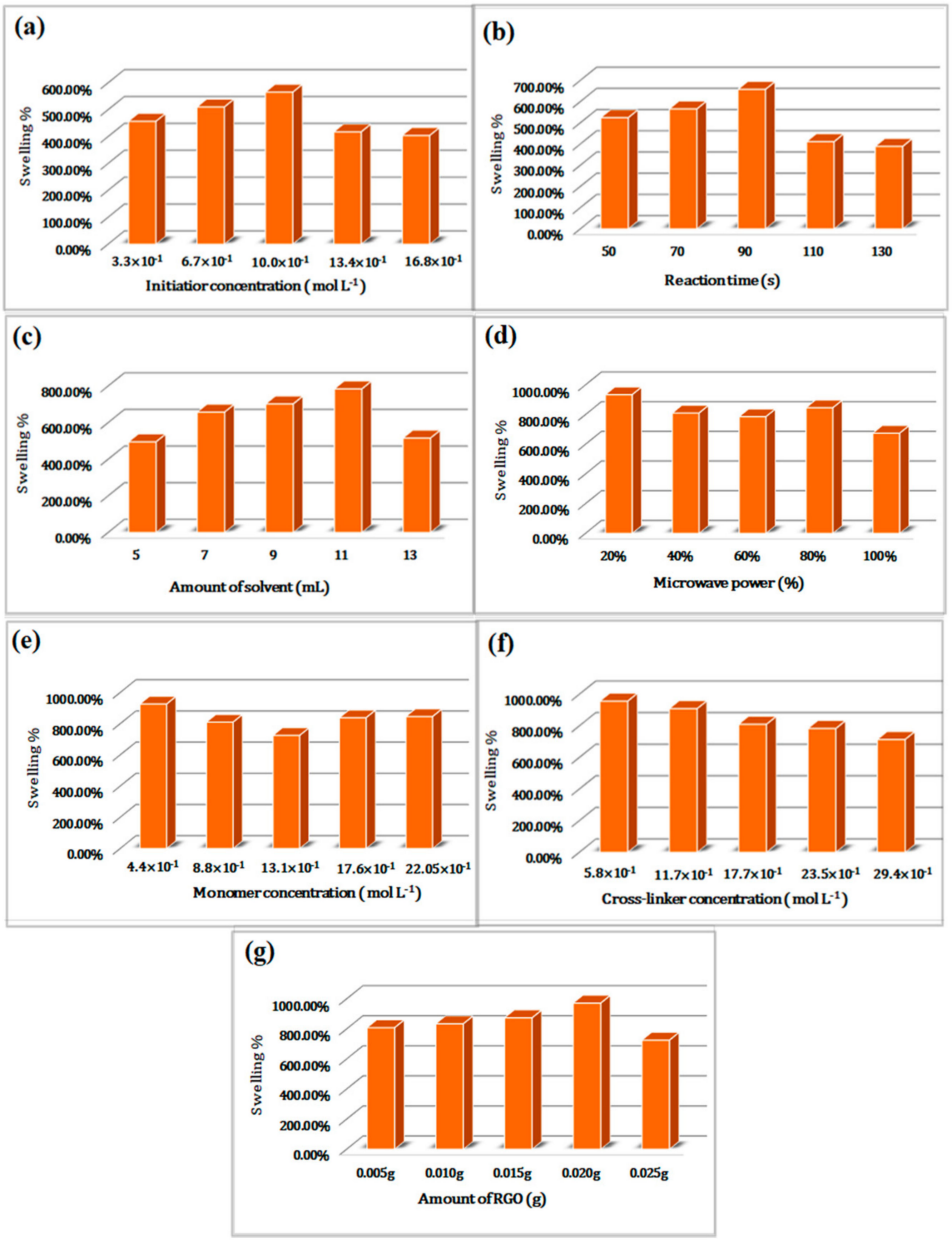
Effect of (**a**) initiator (KPS) concentration, (**b**) reaction time (s), (**c**) amount of solvent (mL), (**d**) microwave power (%), (**e**) monomer (DMA) concentration, (**f**) cross-linker (NMBA) concentration and (**g**) amount of reduced graphene oxide (RGO) on gum tragacanth-cl-*N,N*-dimethylacrylamide hydrogel swelling.

The swelling percentage of GT-cl-poly(DMA) hydrogel was affected by the concentration of KPS and results are shown in Figure 1a. The maximum swelling of 565.5% was observed at 10 × 10^−1^ mol L^−1^ of KPS. Below this concentration (<10 × 10^−1^ mol L^−1^), the swelling percentage was lower due to inadequate initiator, which was unable to produce appropriate active sites on GT-cl-poly(DMA).

#### 3.2.2. Reaction Time

The GT-cl-poly(DMA) hydrogel showed the highest swelling percentage (657.8%) at 90 s (Figure 1b). The swelling percentage was decreased from 90 s to 130 s, this might be due to the formation of excess branched chains that could inhibit the expansion of the polymer.

#### 3.2.3. Solvent

The solvent volume was varied from 5 mL to 13 mL in the formation of GT-cl-poly(DMA) hydrogel (Figure 1c). The maximum swelling (784.4%) was obtained at solvent volume of 11 mL. At higher solvent volume beyond 11 mL, the swelling percentage of GT-cl-poly(DMA) hydrogel was decreased, the excess solvent volume lowered the concentration of KPS, DMA and NMBA resulted in poor degree of polymerization. 

#### 3.2.4. Microwave Power

The swelling percentage was maximum at 20% of microwave power for GT-cl-poly(DMA) hydrogel (Figure 1d). The swelling percentage was lower at microwave power above 20%. This was due to the formation of excess radical led to increase the homo-polymerization rate reaction. Hence, the microwave power was kept at 20% for further synthesis reaction of GT-cl-poly(DMA) hydrogel.

#### 3.2.5. Monomer (DMA) Concentration

The swelling percentage was affected by concentration of monomer (DMA) used in the development of GT-cl-poly(DMA) hydrogel. The maximum swelling percentage was observed at 4.4 × 10^−1^ mol L^−1^ of DMA concentration (Figure 1e). The increase in the concentration of DMA monomer from 4.4 × 10^−1^ mol L^−1^ to 22.05 × 10^−1^ mol L^−1^ led to decrease the swelling percentage due to the self cross-linking of DMA.

#### 3.2.6. Cross-Linker (NMBA)

Figure 1f demonstrates the effect of NMBA concentration (5.8 × 10^−1^–29.4 × 10^−1^ mol L^−1^) on the swelling percentage of GT-cl-poly(DMA) hydrogel. The recorded highest swelling percentage was 957.2% at NMBA concentration of 5.8 × 10^−1^ mol L^−1^. Beyond 5.8 × 10^−1^ mol L^−1^, the excess network was developed due to the more cross-linking points. Thus, the excess network led to decrease the available pores and swelling percentage of GT-cl-poly(DMA) hydrogel.

#### 3.2.7. RGO Loading

Figure 1g shows the influence of RGO loading on the swelling of GT-cl-poly(DMA) hydrogel. The rise in the amount of RGO from 0.005 g to 0.020 g was attributed to an increase in swelling percentage. This was due to an increase in hydrophilic group and surface area of GT-cl-poly(DMA) on the incorporation of RGO. Any further increase in RGO loading (> 0.020 g) was found to decrease the swelling percentage of GT-cl-poly(DMA) hydrogel. This might be attributed to the increased cross-linking density of composite hydrogel networks and the aggregations of excessive RGO in the hydrogel matrix.

### 3.3. FTIR

**Figure 2:**
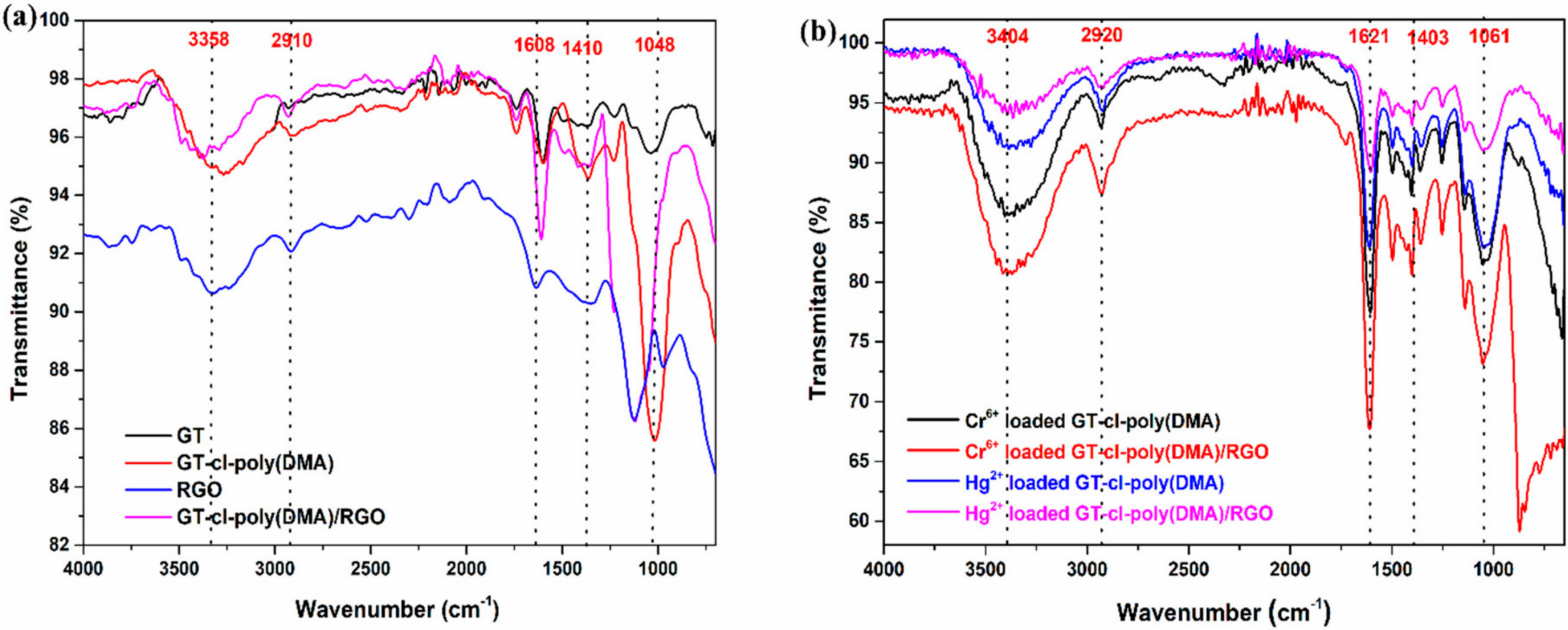
Fourier transform infrared of (**a**) gum tragacanth (GT), gum tragacanth-cl-*N,N*-dimethylacrylamide (GT-cl-poly(DMA)) hydrogel, RGO and reduced graphene oxide incorporated gum tragacanth-cl-*N,N*-dimethylacrylamide (GT-cl-poly(DMA)/RGO) hydrogel composite, (**b**) Hg^2+^ and Cr^6+^ loaded GT-cl-poly(DMA) hydrogel and GT-cl-poly(DMA)/RGO hydrogel composite.

The FTIR graphs of samples are presented in Figure 2. In spectrum of RGO (Figure 2a), broadband of nearly 3358 cm^−1^ can be attributed to –OH stretching mode. The peak at 1421 cm^−1^ corresponds to the carboxylic acid and peak at 1625 cm^−1^ belongs to the –C=C group in the aromatic rings. The peak at 1128 cm^−1^ is due to –C–O stretching in the C–OH functional groups of RGO [29]. The shifting of –C–O stretching from 1128 cm^−1^ to 1125 cm^−1^ in GT-cl-poly(DMA)/RGO hydrogel composite is related to the successful incorporation of RGO in GT-cl-poly(DMA) hydrogel. The bands at 1638 cm^−1^ and 1748 cm^−1^ correspond to asymmetric stretching of the carboxylate group and asymmetric stretching of C=O in galacturonic acid respectively [30], peak at 1142 cm^−1^ ascribed to antisymmetric vibrations of C–O–C linkage in glycosidic groups [31]. The asymmetric stretching of C=O shows shifting of peaks from 1748 cm^−1^ to 1750 cm^−1^ after the crosslinking of poly(DMA). The GT-cl-poly(DMA)/RGO hydrogel composite (Figure 2a) shows shift related to asymmetric stretching of C=O from 1750 cm^−1^ to 1758 cm^−1^ suggesting interaction between RGO and GT-cl-poly(DMA) hydrogel. The peaks at 1608 cm^−1^ and 1410 cm^−1^ in GT-cl-poly(DMA) hydrogel ascribed to stretching vibrations of poly(DMA) amide group [32]. These stretching vibrations of poly(DMA) show peak shifting from 1608 cm^−1^ to 1612 cm^−1^ and from 1410 cm^−1^ to 1403 cm^−1^ in GT-cl-poly(DMA)/RGO hydrogel composite which confirms the changes in the structure of poly(DMA) after RGO incorporation. In GT-cl-poly(DMA)/RGO, the broadband of O–H stretching vibration shifted from 3403 cm^−1^ to 3382 cm^−1^ which may be attributed to the RGO interaction with GT-cl-poly(DMA) through intermolecular hydrogen bonds. The peaks intensity of GT-cl-poly(DMA)/RGO hydrogel composite is slightly lower than the GT-cl-poly(DMA) hydrogel, which also confirms the RGO dispersion in GT-cl-poly(DMA)/RGO hydrogel composite. The absorption band at 2910 cm^−1^ was attributed to stretching vibrations of aliphatic C–H [10]. Also, peaks at 1048 cm^−1^ and 1052 cm^−1^ in the spectra of hydrogels correspond to the –C–O bending. After the adsorption of Hg^2+^ and Cr^6+^ on GT-cl-poly(DMA) hydrogel and GT-cl-poly(DMA)/RGO hydrogel composite, peak due to carboxylate groups was shifted from 1612 cm^−1^ to 1621 cm^−1^ and the intensity of the peaks decreases (Figure 2b). The peaks at 1410 cm^−1^ and 1048 cm^−1^ were shifted to 1403 cm^−1^ and 1061 cm^−1^ respectively, which was probably due to the interactions of metal ions to the active site of adsorbent. The peaks intensity of Hg^2+^ loaded GT-cl-poly(DMA) hydrogel and GT-cl-poly(DMA)/RGO hydrogel composite was lower than the Cr^6+^ loaded GT-cl-poly(DMA) hydrogel and GT-cl-poly(DMA)/RGO hydrogel composite, which supports higher Hg^2+^ adsorption than Cr^6+^ adsorption.

### 3.4. XRD

**Figure 3:**
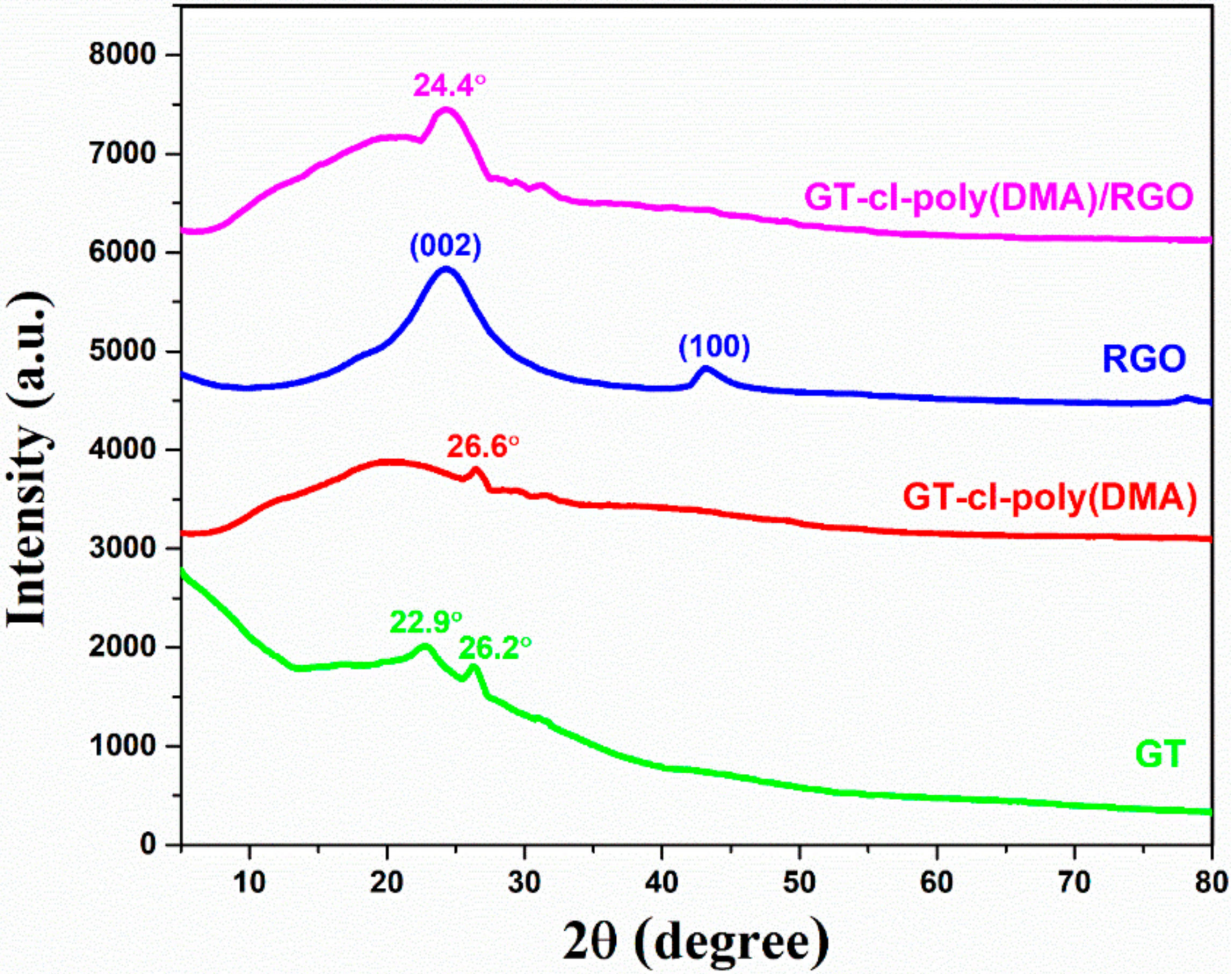
X-ray diffraction pattern for gum tragacanth (GT), gum tragacanth-cl-*N,N*-dimethylacrylamide (GT-cl-poly(DMA)) hydrogel, RGO and reduced graphene oxide incorporated gum tragacanth-cl-*N,N*-dimethylacrylamide (GT-cl-poly(DMA)/RGO) hydrogel composite.

The XRD pattern of GT, gum tragacanth-cl-*N,N*-dimethylacrylamide hydrogel (GT-cl-poly(DMA)), RGO and reduced graphene oxide incorporated gum tragacanth-cl-*N,N*-dimethylacrylamide hydrogel composite (GT-cl-poly(DMA)/RGO) is shown in Figure 3. The RGO formation was confirmed by the characteristic peak at 2θ = 24.3° [33]. On applying Bragg’s law, the calculated interlayer spacing of RGO was 0.367 nm. Another peak of RGO at 2θ = 43.6° corresponded to the fingermark of graphite indicating the regeneration of graphitic onto RGO [34]. According to Scherrer’s formula the calculated particle size of RGO at 2θ = 24.3° was 0.894 nm. In the case of GT, the diffraction peak occurred at 2θ = 22.9° and 26.2°, exhibited semi-crystalline in nature [35]. The slight shifting of the peak at 2θ = 26.6° in XRD pattern of GT-cl-poly(DMA) hydrogel confirms the crosslinking of DMA with polysaccharide by destroying semi-crystalline structure into the amorphous structure. The broad peak in GT-cl-poly(DMA)/RGO hydrogel composite indicates the poor ordered arrangement of RGO in GT-cl-poly(DMA) hydrogel supported by SEM morphology.

### 3.5. SEM

**Figure 4:**
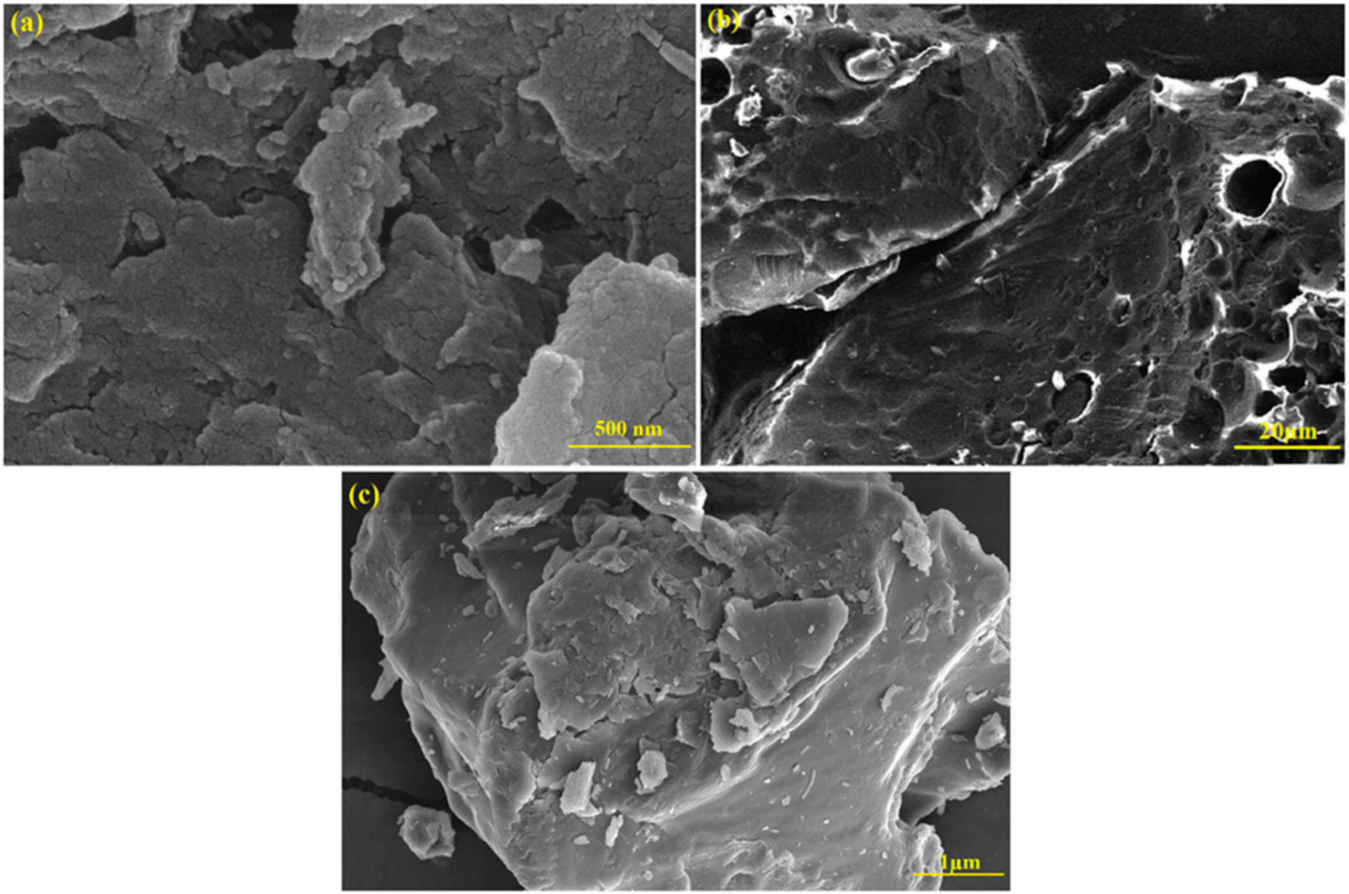
Scanning electron microscope images of (**a**) RGO, (**b**) gum tragacanth-cl-*N,N*-dimethylacrylamide hydrogel and (**c**) reduced graphene oxide incorporated gum tragacanth-cl-*N,N*-dimethylacrylamide hydrogel composite.

Microscopic images of RGO, gum tragacanth-cl-*N,N*-dimethylacrylamide hydrogel and reduced graphene oxide incorporated gum tragacanth-cl-*N,N*-dimethylacrylamide hydrogel composite are shown in Figure 4. In RGO, Figure 4a shows the aggregated wrinkled structure, which means particles were closely associated. The RGO morphology showed the formation of agglomerated RGO with estimated average grain size of 20–25 nm. Figure 4b shows the distribution of certain bulges on a quite smooth, porous and compact surface of gum tragacanth-cl-*N,N*-dimethylacrylamide hydrogel. After the incorporation of RGO, reduced graphene oxide incorporated gum tragacanth-cl-*N,N*-dimethylacrylamide hydrogel composite showed (Figure 4c) the rough and irregular surface with reduced size which was beneficial in fast adsorption of Hg^2+^ and Cr^6+^.

### 3.6. EDS

**Figure 5:**
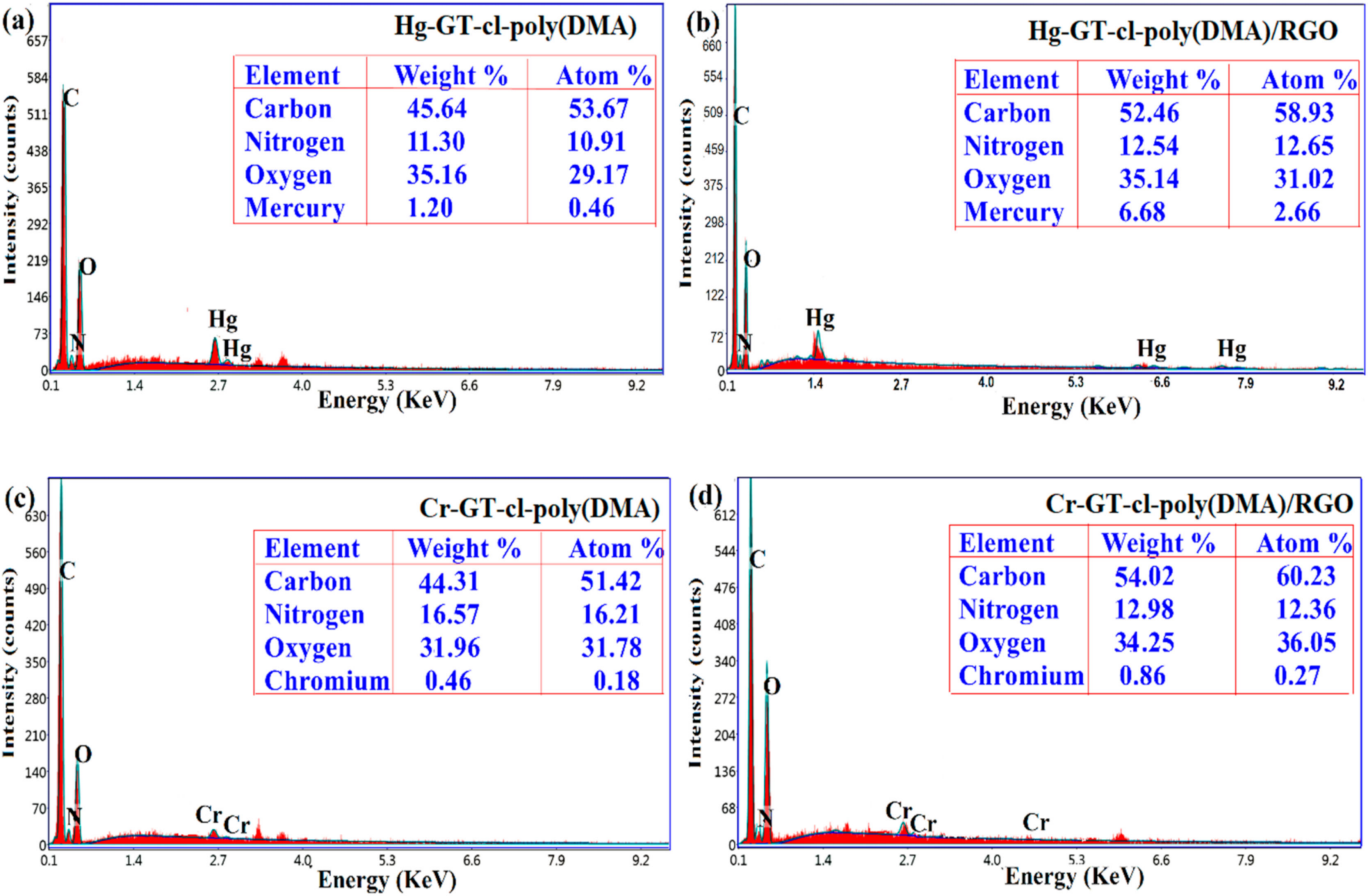
Energy dispersive X-ray spectroscopy and elemental weight percentage for (**a**) Hg^2+^ adsorbed gum tragacanth-cl-*N,N*-dimethylacrylamide (GT-cl-poly(DMA)) hydrogel, (**b**) Hg^2+^ adsorbed reduced graphene oxide incorporated gum tragacanth-cl-*N,N*-dimethylacrylamide (GT-cl-poly(DMA)/RGO) hydrogel composite, (**c**) Cr^6+^ adsorbed gum tragacanth-cl-*N,N*-dimethylacrylamide (GT-cl-poly(DMA)) hydrogel and (**d**) Cr^6+^ adsorbed reduced graphene oxide incorporated gum tragacanth-cl-*N,N*-dimethylacrylamide (GT-cl-poly(DMA)/RGO) hydrogel composite.

The elemental distribution of carbon, oxygen, nitrogen, mercury and chromium in hydrogel matrix was evaluated by EDS analysis and the spectra for Hg^2+^ adsorbed GT-cl-DMA hydrogel, Hg^2+^ adsorbed GT-cl-poly(DMA)/RGO hydrogel composite, Cr^6+^ adsorbed GT-cl-poly(DMA) and Cr^6+^ adsorbed GT-cl-poly(DMA)/RGO are presented in Figure 5. It is evident from the elemental analysis that Hg^2+^ and Cr^6+^ ions were successfully adsorbed by GT-cl-poly(DMA) and GT-cl-poly(DMA)/RGO. Importantly, the weight percentages of Hg^2+^ and Cr^6+^ ions were higher in the case of Hg^2+^ adsorbed GT-cl-poly(DMA)/RGO (6.68%) (Figure 5b) and Cr^6+^ adsorbed GT-cl-poly(DMA)/RGO (0.86%) (Figure 5d) than the Hg^2+^ adsorbed GT-cl-poly(DMA) (1.20%) (Figure 5a) and Cr^6+^ adsorbed GT-cl-poly(DMA) (0.46%) respectively (Figure 5c). Hence, GT-cl-poly(DMA)/RGO hydrogel composite showed better adsorption capability than GT-cl-poly(DMA) hydrogel. Also, the weight percentage of carbon is higher the in case of GT-cl-poly(DMA)/RGO hydrogel composite than the GT-cl-poly(DMA) hydrogel which confirmed the successful dispersion of RGO in GT-cl-poly(DMA) hydrogel matrix.

### 3.7. Application of Gum Tragacanth-cl-N,N-dimethylacrylamide (GT-cl-poly(DMA)) Hydrogel and Reduced Graphene Oxide Incorporated Gum Tragacanth-cl-N,N-dimethylacrylamide (GT-cl-poly(DMA)/RGO) Hydrogel Composite for Removal of Hg^2+^ and Cr^6+^

#### 3.7.1. Influence of RGO Loading on the Removal of Hg^2+^ and Cr^6+^

**Figure 6:**
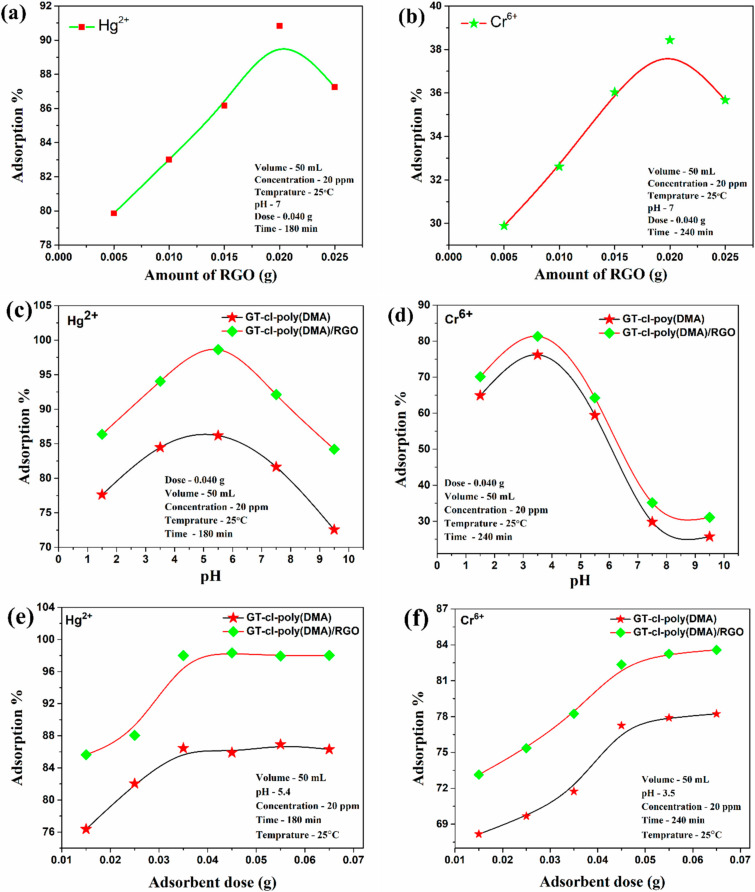
Influence of (**a**) RGO loading on Hg^2+^ adsorption (**b**) RGO loading on Cr^6+^ adsorption, (**c**) pH on adsorption of Hg^2+^, (**d**) pH on adsorption of Cr^6+^, (**e**) adsorbent dose on adsorption of Hg^2+^, (**f**) adsorbent dose on adsorption of Cr^6+^. Adsorbent: gum tragacanth-cl-*N,N*-dimethylacrylamide (GT-cl-poly(DMA)) hydrogel and reduced graphene oxide incorporated gum tragacanth-cl-*N,N*-dimethylacrylamide (GT-cl-poly(DMA)/RGO) hydrogel composite.

In the GT-cl-poly(DMA) hydrogel matrix, different quantities of RGO (0.005 g, 0.01 g, 0.015 g, 0.02 g and 0.025 g) were incorporated to study the effect RGO loading on metal ions removal. The adsorption percentages for without RGO were 70.6% and 20.4% for Hg^2+^ and Cr^6+^ respectively. The adsorption percentages for Hg^2+^ (Figure 6a) and Cr^6+^ (Figure 6b) ions were enhanced on raising the concentration of RGO from 0.005 g to 0.020 g. The RGO contains carboxylic groups which boost interactions with metal ions resulting in high percentage adsorption [30]. The removal efficiency of Hg^2+^ and Cr^6+^ was 90.7% and 38.4% at RGO loading of 0.020 g. The development of tough three-dimensional networks was responsible for the decrease in adsorption percentage at higher RGO loading (>0.020). Therefore, 0.020 g was the optimized dose of RGO in the formation of GT-cl-poly(DMA)/RGO for removal of Hg^2+^ and Cr^6+^.

#### 3.7.2. Influence of pH on Removal of Hg^2+^ and Cr^6+^ by GT-cl-poly(DMA) Hydrogel and GT-cl-poly(DMA)/RGO Hydrogel Composite

**Scheme 2:**
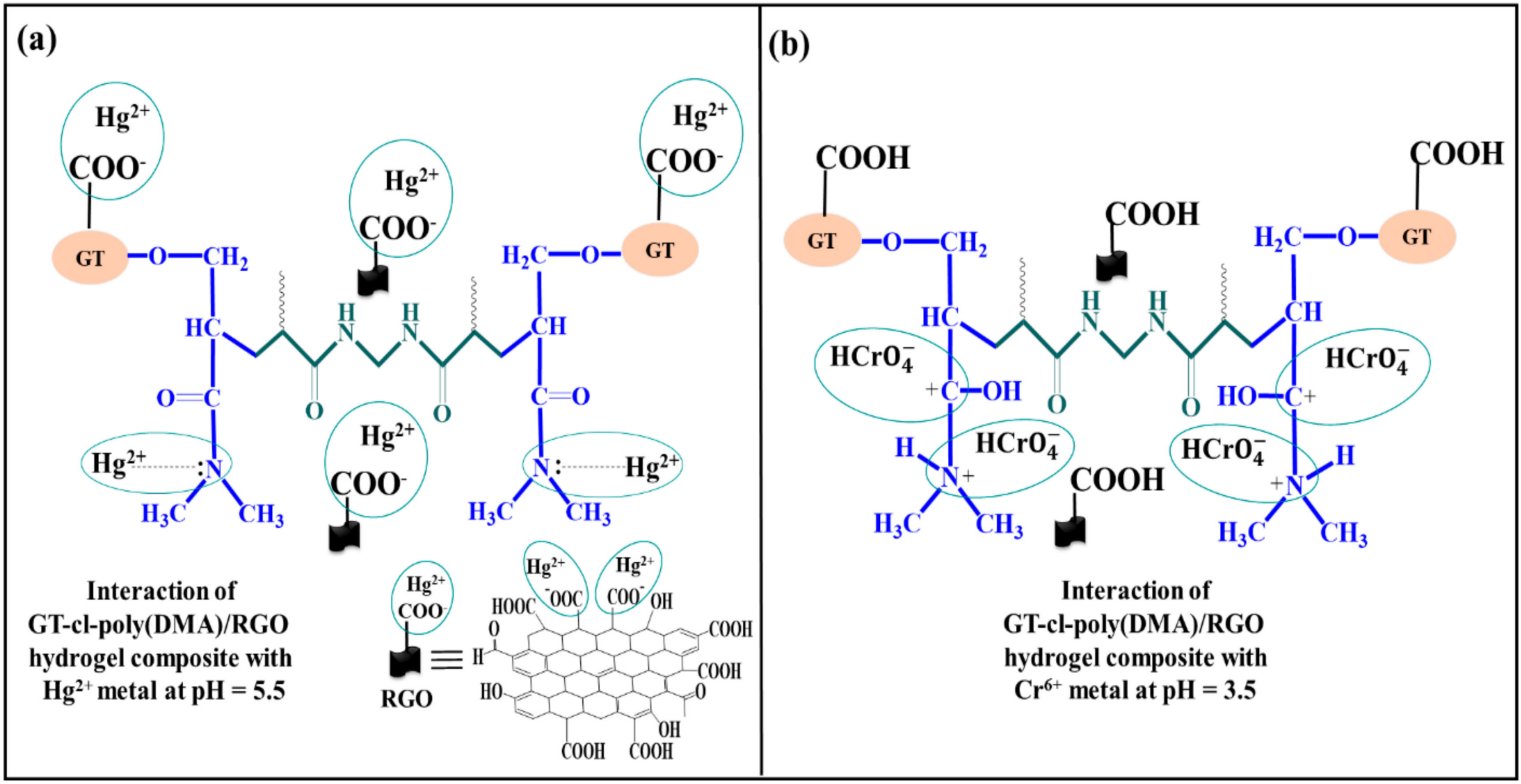
Possible interactions of (**a**) Hg^2+^ at pH 5.5 and (**b**) Cr^6+^ at pH 3.5 with GT-cl-poly(DMA)/RGO hydrogel composite adsorbent.

The impact of pH on adsorption percentage for Hg^2+^ (Figure 6c) and Cr^6+^ (Figure 6d) by gum tragacanth-cl-*N,N*-dimethylacrylamide (GT-cl-poly(DMA)) hydrogel and reduced graphene oxide incorporated gum tragacanth-cl-*N,N*-dimethylacrylamide (GT-cl-poly(DMA)/RGO) hydrogel composite are shown in Figure 6. The removal percentage of Hg^2+^ was first increased from pH 1.5 (77.6% for GT-cl-poly(DMA), 86.3% for GT-cl-poly(DMA)/RGO) to 5.5 (86.1% for GT-cl-poly(DMA), 97.6% for GT-cl-poly(DMA)/RGO) and then decreased from pH 5.5 (86.1%, 97.6%) to 9.5 (72.5%, 84.2%). The reported highest removal efficiencies for Hg^2+^ were 86.1% and 97.6% by GT-cl-poly(DMA) hydrogel and GT-cl-poly(DMA)/RGO) hydrogel composite respectively at 5.5 pH. At low pH, the concentration of H^+^ ions was high which could compete with Hg^2+^ on GT-cl-poly(DMA)/RGO surface resulting in poor binding of Hg^2+^ [36]. However, high pH was responsible for the decrease in the concentration of H^+^ ions in the solution and improves the binding potential of Hg^2+^ ions to the surface of GT-cl-poly(DMA)/RGO) (Scheme 2a). Hence adsorption of Hg^2+^ was increased from pH 1.1 to 5.5. The dominant species were Hg(OH)_2_ and HgCl_4_^2−^ [37] at pH above 5.5. The electrostatic repulsion among Hg(OH)_2_ or HgCl_4_^2−^ [38] and negatively charged GT-cl-poly(DMA)/RGO) was responsible for low Hg^2+^ adsorption percentage at pH above 5.5.

In the case of Cr^6+^, the recorded maximum adsorption percentages were 76.1% and 81.5% (Figure 6d) for GT-cl-poly(DMA) hydrogel and GT-cl-poly(DMA)/RGO hydrogel composite correspondingly at pH 3.5. The dominant Cr^6+^ species [39] are as: H_2_CrO_4_ (pH < 3.5), HCr$O_{4}^{-}$ (pH < 7), ${\mathrm{CrO}}_{4}^{2-}$ (pH > 7). The Cr^6+^ ions were exists in solution as negatively charged ${\mathrm{HCrO}}_{4}^{-}$ at pH 3.5. Therefore, electrostatic attraction of HCr$O_{4}^{-}$ [40] took place at pH 3.5 with protonated positively charged group of GT-cl-poly(DMA)/RGO) (Scheme 2b). Hence, Cr^6+^ exhibited maximum adsorption percentage at 3.5 pH. At pH < 3.5, electrostatic attraction for adsorption was reduced due to the dominance of H_2_CrO_4_. Also, with increasing pH from 3.5 to 7, the protonated group on GT-cl-poly(DMA)/RGO decreases which reduces the electrostatic attraction. At pH > 7, electrostatic repulsion between dominant ${\mathrm{CrO}}_{4}^{2-}$ species [40] and deprotonated GT-cl-poly(DMA)/RGO was attributed to low Cr^6+^ adsorption.

#### 3.7.3. Influence of GT-cl-poly(DMA) Hydrogel and GT-cl-poly(DMA)/RGO Hydrogel Composite Dose for Removal of Hg^2+^ and Cr^6+^

The effect of adsorbents dosages (0.015 – 0.065 g) on the removal of metal ions are represented in Figure 6e,f. The adsorption percentage was increased by increasing the adsorbent dosage. This was due to the existence of more adsorption sites with enhanced dose of adsorbent. The removal efficiencies of Hg^2+^ were found to be 86.4% and 98.4% by GT-cl-poly(DMA) hydrogel and GT-cl-poly(DMA)/RGO hydrogel composite correspondingly at dose of 0.035 g. The reported Cr^6+^ ion removal percentages were 77.2% and 82.3% by using GT-cl-poly(DMA) hydrogel and GT-cl-poly(DMA)/RGO hydrogel composite respectively at optimized dose of 0.045 g. Thus, 0.035 g (for Hg^2+^) and 0.045 g (for Cr^6+^) were the ideal doses used for experiments.

### 3.8. Adsorption Kinetics

The pseudo first-order rate equation is given as:

(3) $$\;\log\left(q_{e}-{\;q}_{t}\right)={\;\mathrm{logq}}_{e}-\frac{K_{1}}{2.303}t$$

where, q_e_ and q_t_ are the adsorption capacity at equilibrium (mg g^−1^) and time t respectively and K_1_ is the pseudo first order kinetics rate constant.

The pseudo second-order rate equation is given as:

(4) $$\frac{t}{q_{t}}=\;\frac{1}{K_{2\;}q_{e}^{2}}+\;\frac{t}{q_{e}}$$

where K_2_ is the pseudo second-order kinetics rate constant.

**Figure 7:**
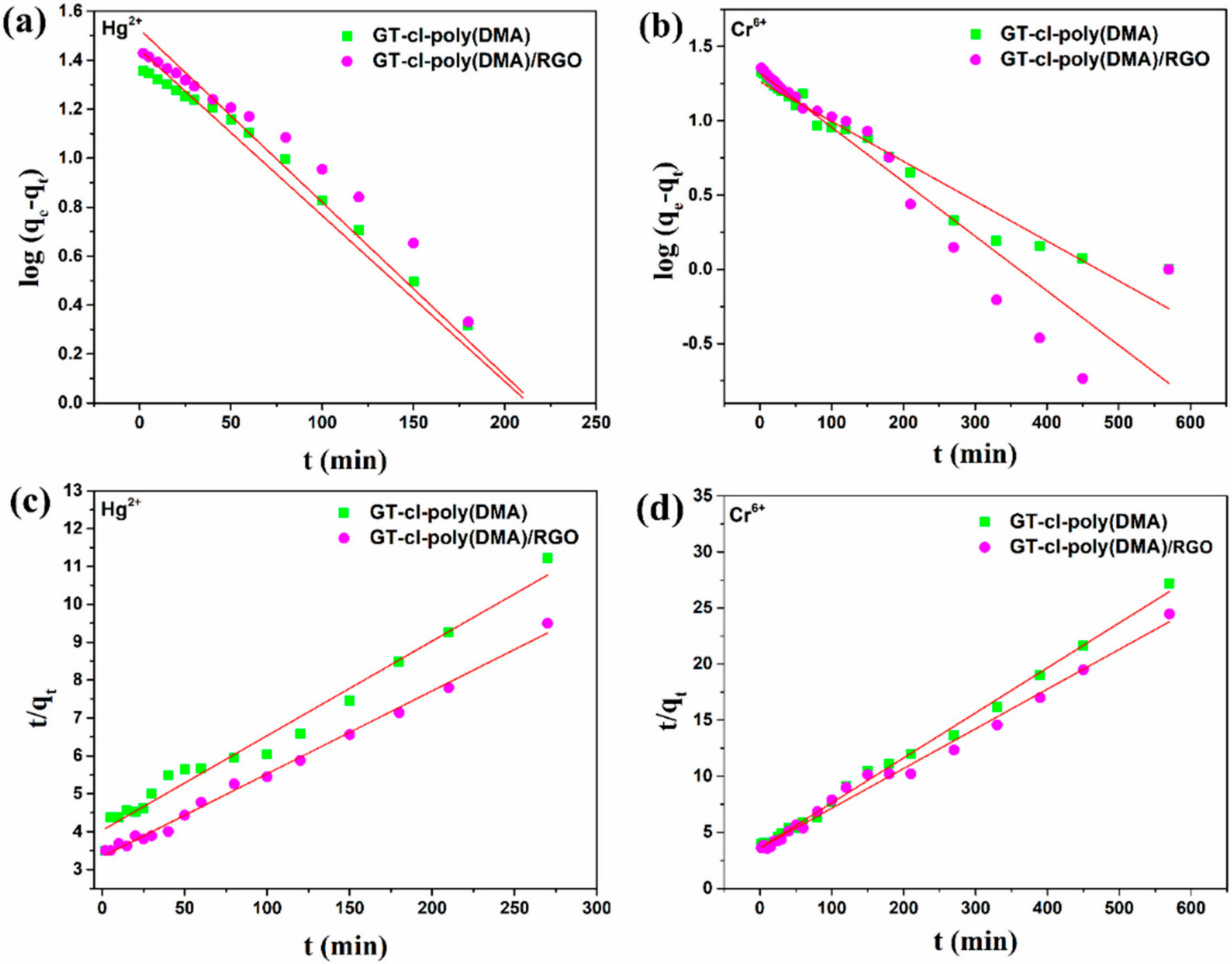
Pseudo first order for (**a**) Hg^2+^ and (**b**) Cr^6+^, pseudo second order for (**c**) Hg^2+^ and (**d**) Cr^6+^. (Experimental conditions for Hg^2+^: adsorbent dose—0.035 g, pH—5.5, metal ion concentration—20 ppm, rpm = 200 and for Cr^6+^: adsorbent dose—0.045 g, pH—3.5, metal ion concentration—20 ppm, rpm = 200).

The removal mechanism for Hg^2+^ and Cr^6+^ by GT-cl-poly(DMA) and GT-cl-poly(DMA)/RGO were solved by different kinetic models as given in Equations (3) and (4). The parameters (pseudo-first-order: R^2^, K_1_, q_e_) were calculated from Figure 7a,b (Table 4). The parameters (pseudo-second-order: K_2_, q_e_) and correlation coefficient (R^2^) were calculated from Figure 7c,d (Table 4). The higher R^2^ values for the pseudo-second-order kinetic model supports Hg^2+^ and Cr^6+^ ions adsorption onto GT-cl-poly(DMA) hydrogel and GT-cl-poly(DMA)/RGO hydrogel composite through the pseudo-second-order kinetic model.

### 3.9. Adsorption Isotherms

The Langmuir model is expressed according to Equation (5) as:

(5) $$\frac{C_{e}}{q_{e}}\;=\;\frac{1}{q_{m}b}+\;\frac{C_{e}}{q_{m}}$$

where C_e_ is the equilibrium concentration of metal ions solution, q_e_ is the amount of equilibrium adsorbed metal ions, q_m_ is maximum adsorption capacity and b is the Langmuir isotherm constant. The separation factor R_L_ of Langmuir isotherm was examined by using Equation (6) as:

(6) $$R_{L}=\frac{1}{1+b\times C_{o}}$$

where C_o_ represent the initial concentration of metal ions. The R_L_ values show the nature of adsorption i.e. irreversible (R_L_ = 0), linear (R_L_ = 1), unfavorable (R_L_ > 1) and favorable (0 < R_L_ < 1). The Freundlich isotherm model is given by Equation (7) as:

(7) $${\mathrm{lnq}}_{e}={\mathrm{lnK}}_{F}+\;\frac{1}{n}{\;\mathrm{lnC}}_{e}$$

where K_F_ and n are Freundlich constants and indicate the adsorption capacity and adsorption intensity of adsorbent respectively.

**Figure 8:**
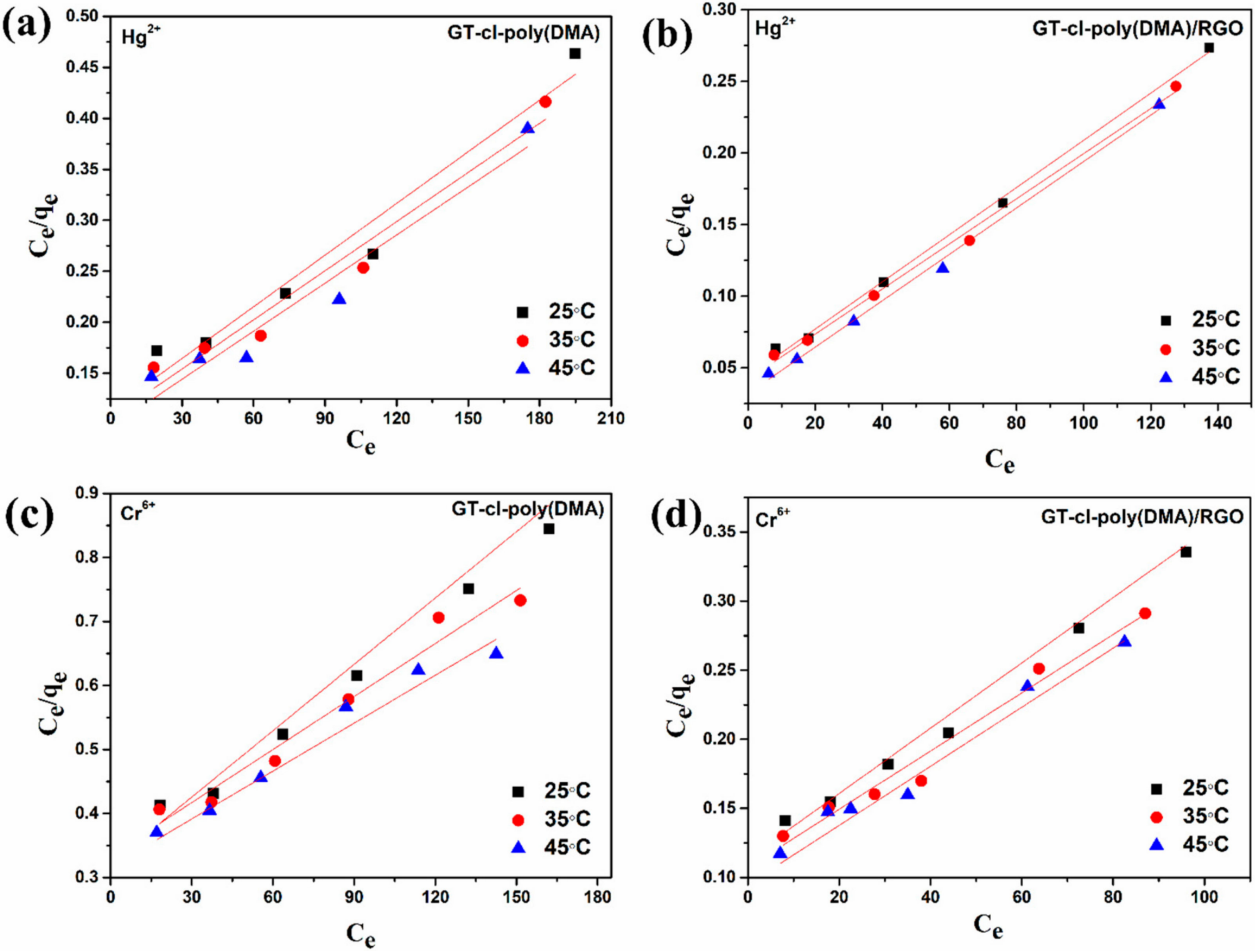
Langmuir isotherm model for Hg^2+^ adsorption by (**a**) gum tragacanth-cl-*N,N*-dimethylacrylamide (GT-cl-poly(DMA)) hydrogel (**b**) reduced graphene oxide incorporated gum tragacanth-cl-*N,N*-dimethylacrylamide (GT-cl-poly(DMA)/RGO) hydrogel composite, Langmuir isotherm model for Cr^6+^ adsorption by (**c**) gum tragacanth-cl-*N,N*-dimethylacrylamide (GT-cl-poly(DMA)) hydrogel (**d**) reduced graphene oxide incorporated gum tragacanth-cl-*N,N*-dimethylacrylamide (GT-cl-poly(DMA)/RGO) hydrogel composite. (Experimental conditions for Hg^2+^: adsorbent dose—0.035 g, pH—5.5, metal ion concentration—20–300 ppm, rpm = 200 and for Cr^6+^: adsorbent dose—0.045 g, pH—3.5, metal ion concentration—20–500 ppm, rpm = 200).

**Figure 9:**
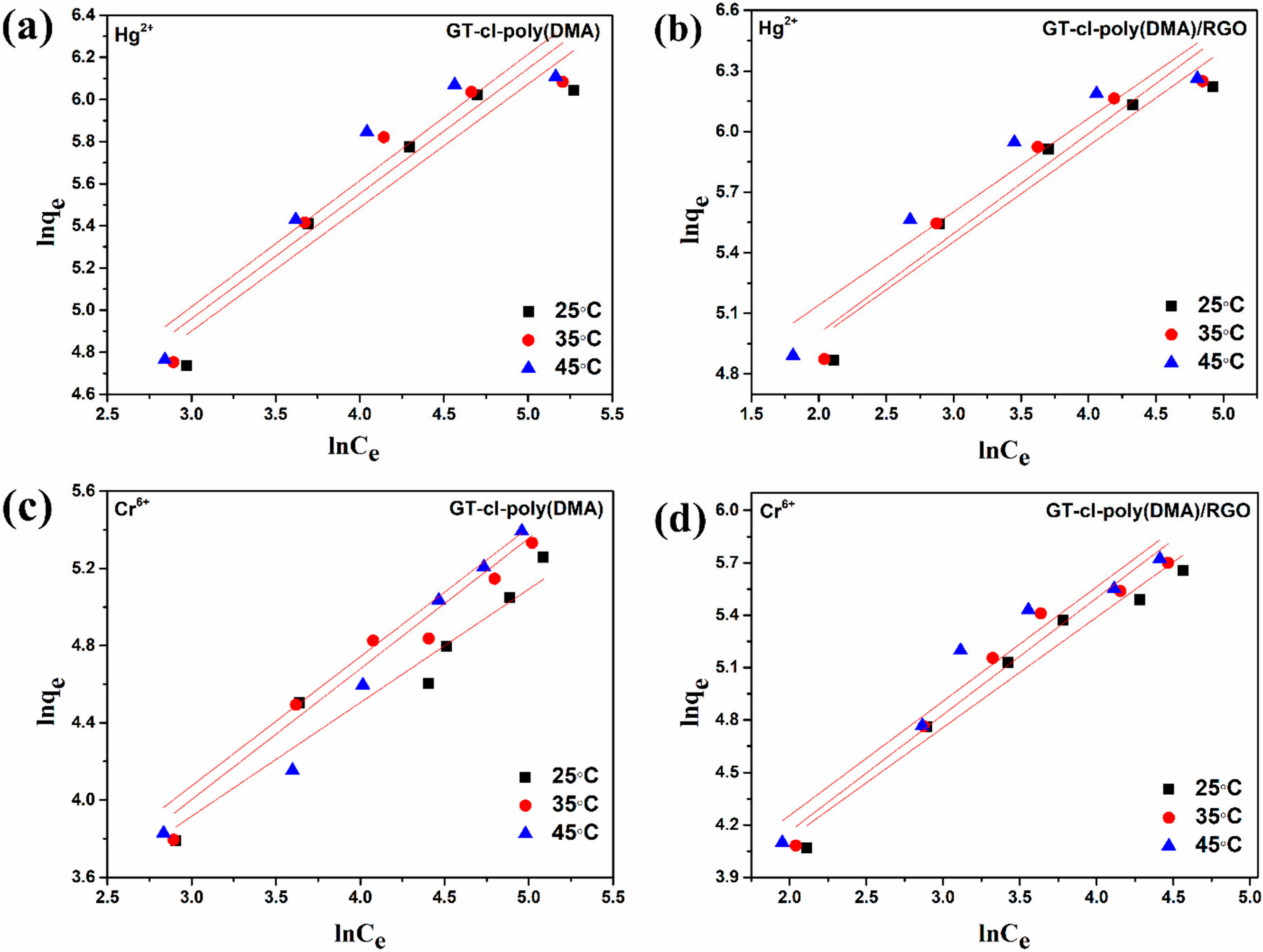
Freundlich isotherm model for Hg^2+^ adsorption by (**a**) gum tragacanth-cl-*N,N*-dimethylacrylamide (GT-cl-poly(DMA)) hydrogel (**b**) reduced graphene oxide incorporated gum tragacanth-cl-*N,N*-dimethylacrylamide (GT-cl-poly(DMA)/RGO) hydrogel composite, Freundlich isotherm model for Cr^6+^ adsorption by (**c**) gum tragacanth-cl-*N,N*-dimethylacrylamide (GT-cl-poly(DMA)) hydrogel (**d**) reduced graphene oxide incorporated gum tragacanth-cl-*N*,*N*-dimethylacrylamide (GT-cl-poly(DMA)/RGO) hydrogel composite, (Experimental conditions for Hg^2+^: adsorbent dose—0.035 g, pH—5.5, metal ion concentration—20−300 ppm, rpm = 200 and for Cr^6+^: adsorbent dose—0.045 g, pH—3.5, metal ion concentration—20−500 ppm, rpm = 200).

The interaction between adsorbent (GT-cl-poly(DMA) hydrogel and GT-cl-poly(DMA)/RGO hydrogel composite) and adsorbate (Hg^2+^ and Cr^6+^) was explained through isotherms model Equations (5) and (7). The Langmuir parameters were calculated from the graph between C_e_/q_e_ and C_e_ (Figure 8a–d) and presented in Table 5. The Freundlich parameters were determined from the graph of lnq_e_ vs lnC_e_ (Figure 9a–d) and depicted in Table 5. For the Langmuir isotherm, the higher R^2^ suggests that the Langmuir isotherm was best suited for the removal of Hg^2+^ and Cr^6+^ ions on GT-cl-poly(DMA) hydrogel and GT-cl-poly(DMA)/RGO hydrogel composite. For Hg^2+^, GT-cl-poly(DMA) and GT-cl-poly(DMA)/RGO showed higher removal capacity of 625 mg g^−1^ and 666.6 mg g^−1^ respectively. Similarly, for Cr^6+^, the maximum reported removal capacities were 401.6 mg g^−1^ and 473.9 mg g^−1^ by GT-cl-poly(DMA) hydrogel and GT-cl-poly(DMA)/RGO hydrogel composite respectively.

### 3.10. Relationship between the Adsorption and Swelling

**Figure 10:**
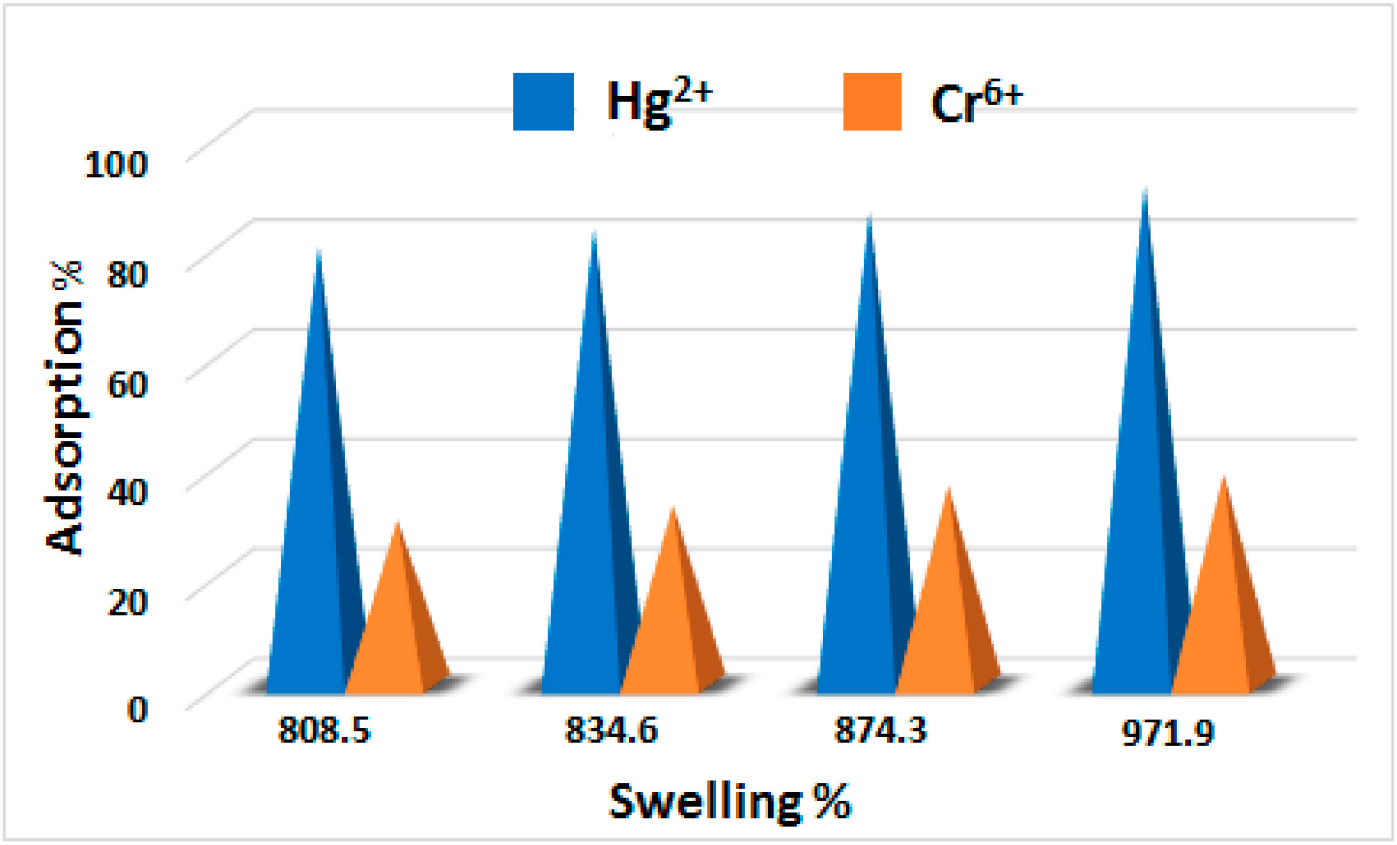
Adsorption percentage and swelling percentage of reduced graphene oxide incorporated gum tragacanth-cl-*N,N*-dimethylacrylamide (GT-cl-poly(DMA)/RGO) hydrogel composite.

For the investigation of the correlation between the swelling of GT-cl-poly(DMA)/RGO hydrogel composite and adsorption of the Hg^2+^ and Cr^6+^ onto GT-cl-poly(DMA)/RGO hydrogel composite, the adsorption percentage was determined using aqueous metal solution (20 mg L^−1^) and the swelling experiments were performed in distilled water. The relationship between the adsorption values versus swelling values is presented in Figure 10. It is clear from Figure 10 that the adsorption percentage is directly proportional to the swelling percentage of the adsorbent. The adsorption percentages for Hg^2+^ and Cr^6+^ were increased from 78.9% to 90.7% and 29.8% to 38.4% respectively when the swelling percentage of GT-cl-poly(DMA)/RGO rise from 834.6% to 971.9%.

### 3.11. Adsorption-Desorption Study

**Figure 11:**
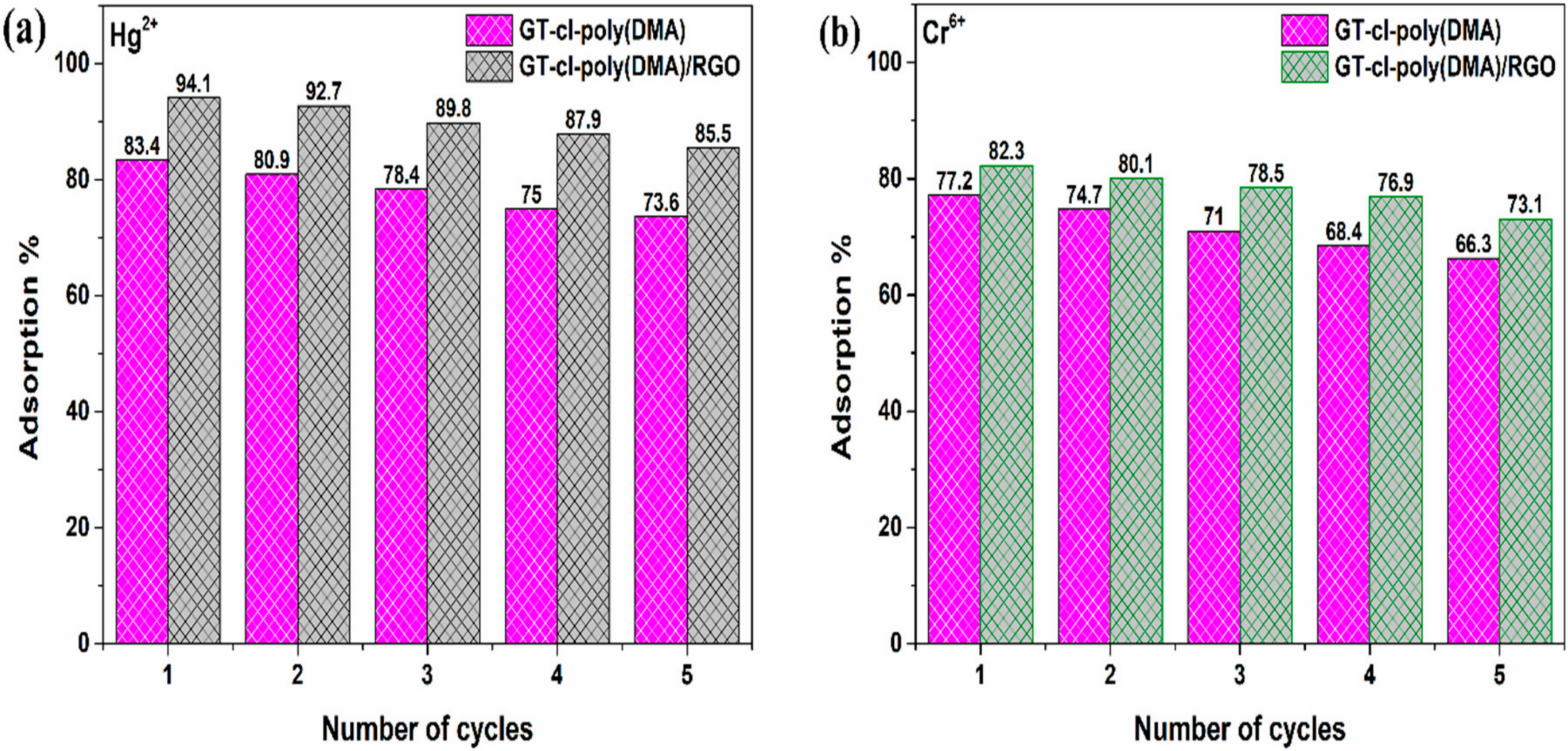
Recycling ability of gum tragacanth-cl-*N,N*-dimethylacrylamide (GT-cl-poly(DMA)) hydrogel and reduced graphene oxide incorporated gum tragacanth-cl-*N,N*-dimethylacrylamide (GT-cl-poly(DMA)/RGO) hydrogel composite for the removal of (**a**) Hg^2+^ and (**b**) Cr^6+^ metal ions up to five cycles.

For an ideal adsorbent, ability for regeneration without considerable loss of removal percentage is of paramount importance. The five cycles of adsorption-desorption and their effects on percentage adsorption are presented in Figure 11. The Hg^2+^ ions adsorption percentages were 83.4% (1st cycle), 80.9% (2nd cycle), 78.4% (3rd cycle), 75% (4th cycle), 73.6% (5th cycle) and 94.1% (1st cycle), 92.7% (2nd cycle), 89.8% (3rd cycle), 87.9% (4th cycle), 85.5% (5th cycle) for GT-cl-poly(DMA) hydrogel and GT-cl-poly(DMA)/RGO hydrogel composite respectively (Figure 11a). For Cr^6+^, GT-cl-poly(DMA) and GT-cl-poly(DMA)/RGO exhibited 77.2% (1st cycle), 74.7% (2nd cycle), 71% (3rd cycle), 68.4% (4th cycle), 66.3% (5th cycle) and 82.3% (1st cycle), 80.1% (2nd cycle), 78.5% (3rd cycle), 76.9% (4th cycle), 73.1% (5th cycle) respectively (Figure 11b). Hence, the synthesized GT-cl-poly(DMA) hydrogel and GT-cl-poly(DMA)/RGO hydrogel composite can be effectively reused for up to five cycles, which leads to reduction in cost.

## 4. Conclusions

We developed novel reduced graphene oxide incorporated gum tragacanth-cl-*N,N*-dimethylacrylamide (GT-cl-poly(DMA)/RGO) hydrogel composite as reusable adsorbent for Hg^2+^ and Cr^6+^ ions. The reported maximum swelling percentage was 971.9% for reduced graphene oxide incorporated gum tragacanth-cl-*N,N*-dimethylacrylamide hydrogel composite at optimized synthesis conditions (KPS concentration: 10.0 × 10^−1^ mol L^−1^, solvent: 11 mL, reaction time: 90 s, microwave power: 20%, DMA concentration: 4.4 × 10^−1^ mol L^−1^, NMBA concentration: 5.8 × 10^−1^ mol L^−1^ and amount of RGO: 0.020 g). The adsorption efficiencies of 99% and 82% were reported for Hg^2+^ and Cr^6+^ by using GT-cl-poly(DMA)/RGO hydrogel composite at optimized adsorption conditions (for Hg^2+^, pH: 5.5, adsorbent dose: 0.035 g, RGO loading: 0.020 g, Hg^2+^ concentration: 20 ppm, Hg^2+^ volume: 50 mL, time: 270 min, temperature: 25 °C and for Cr^6+^, pH: 3.5, adsorbent dose: 0.045 g, RGO loading: 0.020 g, Cr^6+^ concentration: 20 ppm, Cr^6+^ volume: 50 mL, time: 570 min, temperature: 25 °C). The Q_max_ of Hg^2+^ and Cr^6+^ onto reduced graphene oxide incorporated gum tragacanth-cl-*N,N*-dimethylacrylamide hydrogel composite were 666.6 mg g^−1^ and 473.9 mg g^−1^ correspondingly, which were higher than the Q_max_ of gum tragacanth-cl-*N,N*-dimethylacrylamide hydrogel (Hg^2+^ = 625 mg g^−1^, Cr^6+^ = 401.6 mg g^−1^). The Hg^2+^ and Cr^6+^ adsorption were better depicted through pseudo-second-order and Langmuir isotherm. The gum tragacanth-cl-*N,N*-dimethylacrylamide and reduced graphene oxide incorporated gum tragacanth-cl-*N,N*-dimethylacrylamide adsorbents can be effectively reused for up to five cycles for adsorption of Hg^2+^ and Cr^6+^ ions. Thus, developed adsorbents are highly efficient in heavy metal ion adsorption and can be exploited for environmental remediation application.

## Figures and Tables

**Table 1:** Comparison of different adsorbents with gum tragacanth-cl-*N,N*-dimethylacrylamide (GT-cl-poly(DMA)) hydrogel and reduced graphene oxide incorporated gum tragacanth-cl-*N,N*-dimethylacrylamide (GT-cl-poly(DMA)/RGO) hydrogel composite for adsorption of Hg^2+^ and Cr^6+^ metal ions.

Serial Number	Adsorbent	Hg^2+^ Adsorption Capacity (mg g^−1^)	Cr^6+^ Adsorption Capacity (mg g^−1^)	References
1.	Poly(allylamine-co-methacrylamide-co-dimethylthiourea)	198.23	-	[16]
2.	Sulfhydryl-functional paramagnetic solid-phase adsorbent	51.32	-	[17]
3.	Diethylenetriaminepentaacetic acid-modified cellulose adsorbent	476.2	-	[18]
4.	Cross-linked magnetic chitosan-phenylthiourea resin	135.5	-	[19]
5.	Carboxyl methylcellulose and chitosan-derived nanostructured	-	347.0	[20]
6.	Carboxymethyl cellulose–stabilized sulfidated nano zerovalent iron	-	355.9	[21]
7.	Fungal strain (Rhizopus sp.)	-	9.95	[22]
8.	Surfactant-modified Auricularia auricula spent substrate	-	21.74	[23]
9.	GT-cl-poly(DMA) hydrogel and GT-cl-poly(DMA)/RGO hydrogel composite	636.94 and 666.66	-	Present work
10.	GT-cl-poly(DMA) hydrogel and GT-cl-poly(DMA)/RGO hydrogel composite	-	416.66 and 476.19	Present work

**Table 2:** Comparative analysis for swelling percentage of hydrogels.

Serial Number	Sample	Synthetic Route	Time for Synthesis (s)	Swelling Percentage (%)	References
1.	Carboxymethyl cellulose-cl-poly(lactic acid- co-itaconic acid) hydrogel	Microwave assisted method	90 s	332%	[24]
2.	Tragacanth gum-g-poly(itaconic acid) hydrogel	Microwave-assisted method	220 s	800%	[25]
3.	Chitosan-polyethylene glycol hydrogel membrane	Microwave assisted method	120 s	96.4%	[26]
4.	IPN [(GcA-coll)-cl-poly (AAm-ip-AA)]	Microwave assisted method	150 s	382.1%	[27]
5.	GT-cl-poly(DMA) hydrogel	Microwave assisted method	90 s	957.2%	Present work
6.	GT-cl-poly(DMA)/RGO hydrogel composite	Microwave assisted method	90 s	971.9%	Present work

**Table 3:** Optimized quantities used for the preparation of hydrogels.

Serial Number	Sample Name	GT (g)	KPS (g)	DMA (mL)	NMBA (g)	Solvent (mL)	RGO (g)	Swelling %
1.	GT-cl-poly(DMA) hydrogel	0.500	0.030	0.5	0.030	11	-	957.2%
2.	GT-cl-poly(DMA)/RGO hydrogel composite	0.500	0.030	0.5	0.030	11	0.020	971.9%

**Table 4:** Kinetics model parameters for Hg^2+^ and Cr^6+^ removal by GT-cl-poly(DMA) hydrogel and GT-cl-poly(DMA)/RGO hydrogel composite.

Kinetic Model	Parameters	GT-cl-poly(DMA)		GT-cl-poly(DMA)/RGO	
		Hg^2+^	Cr^6+^	Hg^2+^	Cr^6+^
Pseudo-first-order kinetics	R^2^	0.946	0.931	0.909	0.863
	q_e_ (cal)	25.7	19.0	33.5	21.0
	q_e_ (exp)	28.2	22.6	40.8	25.9
	k_1_	0.011	0.008	0.016	0.006
Pseudo-second-order kinetics	R^2^	0.989	0.995	0.994	0.989
	q_e_ (cal)	29.4	25	45.8	28.3
	q_e_ (exp)	28.2	22.6	40.8	25.9
	k_2_	5.90	4.42	1.42	3.44

**Table 5:** Isotherm model parameters for Hg^2+^ and Cr^6+^ adsorption by GT-cl-poly(DMA) hydrogel and GT-cl-poly(DMA)/RGO hydrogel composite.

Isotherm Models	Temperature	Parameters	GT-cl-poly(DMA)		GT-cl-poly(DMA)/RGO	
			Hg^2+^	Cr^6+^	Hg^2+^	Cr^6+^
Langmuir	25 °C	q_m_ (mg g^−1^)	591.7	289.8	628.9	423.7
		b (L mg^−1^)	0.014	0.010	0.036	0.020
		R_L_	0.405–0.121	0.839–0.370	0.217–0.052	0.856–0.339
		R^2^	0.987	0.964	0.996	0.989
	35 °C	q_m_ (mg g^−1^)	621.1	362.3	662.2	467.2
		b (L mg^−1^)	0.015	0.008	0.035	0.019
		R_L_	0.400–0.119	0.874–0.452	0.220–0.054	0.869–0.371
		R^2^	0.960	0.962	0.994	0.989
	45 °C	q_m_ (mg g^−1^)	625	401.6	666.6	473.9
		b (L mg^−1^)	0.016	0.007	0.046	0.022
		R_L_	0.385–0.112	0.887–0.438	0.167–0.039	0.864–0.353
		R^2^	0.963	0.956	0.995	0.9623
Freundlich	25 °C	K_F_ (mg g^−1^)	1.144	0.765	1.393	1.05
		n	1.72	1.70	2.12	1.58
		R^2^	0.889	0.898	0.905	0.955
	35 °C	K_F_ (mg g^−1^)	1.156	0.679	1.389	1.038
		N	1.69	1.47	2.04	1.52
		R^2^	0.891	0.895	0.914	0.955
	45 °C	K_F_ (mg g^−1^)	1.168	0.726	1.438	1.080
		n	1.66	1.49	2.17	1.53
		R^2^	0.868	0.876	0.898	0.924
